# Optimizing optical coherence tomography to detect occult spermatozoa in rat testis after induced non-obstructive azoospermia

**DOI:** 10.1117/1.JBO.30.4.046005

**Published:** 2025-04-08

**Authors:** Luyang Yu, Yanhe Lue, Hang Yang, Junze Liu, Carlos Vega, Kevin Ho, Jacob Rajfer, Christina Wang, Ronald Swerdloff, B. Hyle Park

**Affiliations:** a University of California Riverside, Department of Bioengineering, Riverside, California, United States; b The Lundquist Institute at Harbor-UCLA Medical Center, Division of Endocrinology, Department of Medicine, Torrance, California, United States; c David Geffen School of Medicine at UCLA, Department of Urology, Los Angeles, California, United States

**Keywords:** non-obstructive azoospermia, optical coherence tomography, micro-TESE, occult sperm in the testis

## Abstract

**Significance:**

The ability to detect and localize sperm in the testes is crucial for the treatment of non-obstructive azoospermia (NOA), a condition where sperm retrieval is challenging due to the lack of visible sperm. Enhancing the accuracy and efficiency of sperm detection can significantly improve the outcomes of microdissection testicular sperm extraction (micro-TESE) procedures in NOA patients.

**Aim:**

We aim to use optical coherence tomography (OCT) to detect the presence or absence of sperm in the imaged areas of the testes and to localize sperm-containing seminiferous tubules in a rat model of NOA.

**Approach:**

Volumetric OCT scanning was performed on 180 distinct regions from the testes of two control and 15 busulfan-treated rats to mimic NOA. Following scanning, excised tubules were observed under a dissecting microscope with transillumination to confirm the presence of sperm. The OCT data were processed by first delineating the tubule lumen and then evaluating the calibrated intensity and attenuation coefficient within the lumen. These quantifications, along with outer tubule diameter, were evaluated to identify sperm by comparison with the results of the microscope examination.

**Results:**

Our OCT results revealed a significant correlation between the presence of sperm and high attenuation coefficients in a rat model of NOA. The accuracy of sperm detection by OCT is 97.8% when compared with microscopic identification. In addition, OCT data were utilized for color-coded processing to automatically distinguish regions with a greater likelihood of the presence of sperm, which may assist surgeons in locating occult sperm in NOA patients.

**Conclusions:**

By providing high-resolution, non-invasive, automatic capture, and color-coded images, OCT has the potential to significantly enhance the efficiency of identification of tubules with spermatozoa during micro-TESE.

## 1 Introduction

Although non-obstructive azoospermia (NOA) is characterized by severe impairment of spermatogenesis and a lack of sperm (spermatozoa) in the ejaculate, focal pockets of sperm have been found in 30% to 60% of affected testes.^1^ Microdissection testicular sperm extraction (micro-TESE)^2^^,^^3^ is the current standard procedure to retrieve occult sperm with a higher sperm retrieval rate and lower degree of postoperative complication than other surgical options such as simple TESE, multiple TESE, biopsy gun needle biopsy, and needle aspiration.^4^^,^^5^ Micro-TESE relies on visual examination through a surgical microscope to identify individual tubules likely to contain sperm based only on cues visible from the outer surface of the tubule, such as diameter and opacity.^3^ The success heavily relies on the experience of the surgeon. Technological advances capable of providing useful information regarding the entire tubule would allow for more informed intra-surgical extraction of tubules.

Previous studies have demonstrated the potential for various technologies to provide such information. Ultrasonically actuated silicon microprobes have been employed to accurately quantify tubule diameters for the identification of regions with the larger diameter tubules of interest but require direct contact between the microprobe and tubule of interest.^6^^,^^7^ Elastic single-scattering spectroscopy does not require contact to optically detect spermatogenesis but does not currently provide this information in a spatially specific manner within a given field of view.^8^ Narrow-band imaging has been demonstrated to identify areas with spermatogenic activity in a label-free manner indirectly through visualization of blood vasculature.^9^ Confocal fluorescence microscopy has been used for more direct in vivo identification of sperm in an animal model of NOA but requires the introduction of fluorescently tagged antibodies.^10^ Multiphoton microscopy can differentiate normal from abnormal spermatogenesis in a label-free manner, the limited lateral field of view (∼1  mm), imaging depth (∼1  mm), and working distance (∼10  mm) make intraoperative use difficult.^11^^,^^12^

A limited number of studies have demonstrated the potential for optical coherence tomography (OCT) to provide cross-sectional imaging of tissue microstructure without the need for exogenous contrast agents. The initial application of OCT imaging to human testicular tissue by Brezinski et al. demonstrated its capability to visualize individual seminiferous tubules and the potential for in vivo biopsy.^13^ Since then, advancements have led to improvements in resolution and imaging depth. A commercial time-domain OCT system with an axial resolution of 10 to 20  μm was applied to the human testis and showed similar seminiferous tubule diameter between OCT intensity images (218  μm) and histological findings (212  μm) in patients with normal spermatogenesis.^14^ More recently, advancements in OCT systems, such as a full-field OCT system with superior axial resolution of 1  μm, provided clear visualization of tubular structures and sperm tails in four normal rats and four busulfan-treated rats, although with a limited depth of view (200  μm to 1 mm).^15^ In addition, two commercial spectral-domain OCT systems have been employed in a bovine model, allowing for the identification of tubule lumens, germ cells, interstitial tissue, and the localization of sperm within tissue samples from OCT intensity images.^16^

Although these seminal studies point toward the potential for OCT to facilitate the identification of sperm-bearing tubules, our goal here is to take a step toward potential future clinical application through a systematic study in an animal model. Although there are a number of differences between human and rat testes,^17^ busulfan treatment in rats through injection^18^ is an established animal model for NOA^10^^,^^15^^,^^19^ and was used for this study. A critical step toward this goal is to more fully utilize the information acquired through OCT. For example, a recent publication demonstrated the ability to quantify epididymal contraction frequency using time-sequenced OCT acquisition.^20^^,^^21^ In this study, we used elevated OCT backscattering intensity localized to sperm^16^ to identify sperm-bearing tubules by considering depth-resolved optical attenuation,^22^ a measure of the rate at which tissue reduces light intensity, in addition to the more traditional measure of back-reflected intensity provided by OCT. We identified distinct optical and structural characteristics capable of distinguishing the tunica, tubule walls, and the lumen of tubules that do and do not contain sperm in a rat model for NOA. We further demonstrate that computationally efficient OCT-based measures can potentially be used to identify single seminiferous tubules containing sperm.

## 2 Materials and Methods

### 2.1 Animal Model

We purchased 17 young adult (6 weeks old) male Sprague–Dawley rats from Charles River Laboratories and housed them in a standard animal facility under controlled temperature (22°C) and photoperiod of 12 h of light and 12 h of darkness with free access to food and water. The animal use protocol (IACUC Project 31940-01) was reviewed and approved by the Institutional Animal Care and Use Committee of The Lundquist Institute at Harbor-UCLA Medical Center. Two of the animals served as controls. NOA was induced in other 15 rats by intraperitoneal injection of busulfan (10  mg/kg) on day 1 and day 24 to eliminate differentiating germ cells while maintaining spermatogonial stem cells (SSCs).^23^ Three to 4 months after the last injection, some of the residual SSCs re-initiate spermatogenesis along the seminiferous tubules that mimic the pathology of NOA testes in men.

### 2.2 Spectral-domain OCT System

**Fig. 1:**
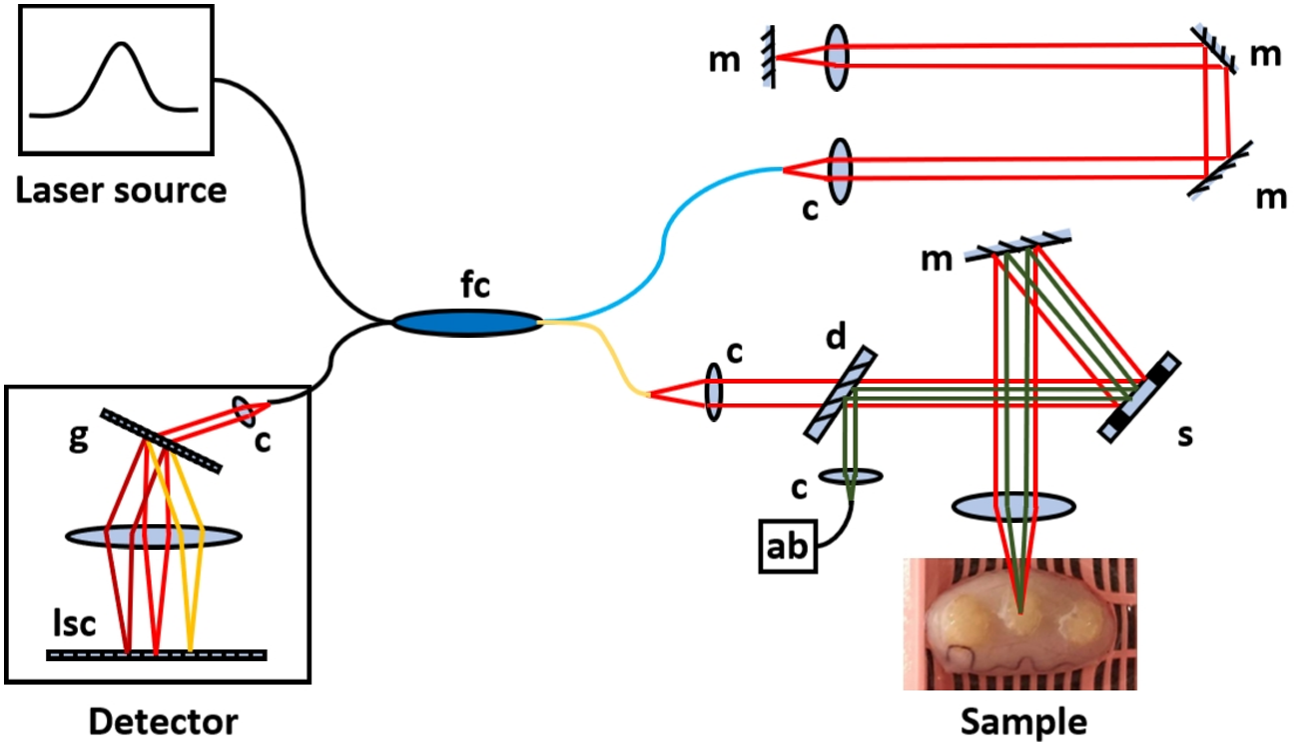
Schematic of the OCT system used in this study, in which light (Superlum cBLMD-T-850-HP-I, λc=850  nm, FWHM bandwidth=165  nm) is split between reference (blue) and sample (gold) arms with a fiber coupler (fc) (75/25). The lateral position of the OCT beam is scanned over a sample using a micro-electro-mechanical systems (MEMS) scanner (Mirrorcle Technologies, A8L2.2-5000AL-TINY48.4-B/F/2TP). Light reflecting back from both arms is recombined and detected on a spectrometer based on a diffraction grating (g) and line scan camera (lsc), and an aiming beam (ab) is incorporated providing visual guidance.

We designed a spectral-domain OCT system specifically for this study (Fig. 1). Based on previously demonstrated evidence that low levels (4 to $10\;J/{\mathrm{cm}}^{2}$) of near-infrared (830 nm) light is not only safe for sperm but can actually increase sperm motility,^24^^,^^25^ our system utilizes a broadband laser in a similar wavelength range [center wavelength 850 nm with a full width at half maximum (FWHM) bandwidth of 165 nm]. OCT light is directed onto a sample at a working distance in excess of 9 cm with a lateral imaging resolution of 20  μm and an incident power of 1.4 mW. It would then require 11.4 msec to irradiate a specific location in a sample at the lower end of the previously cited range ($4\;J/{\mathrm{cm}}^{2}$). Given the imaging scan parameters in a later paragraph, samples are exposed to a far lower level of light irradiation. Detection optics provide depth profiles of 1024 points with a measured axial resolution of 3.1  μm over 1.85 mm of imaging depth. System sensitivity was 104 dB at a line acquisition rate of 8 kHz, with a 10 dB drop over 1 mm.

A number of studies^26^^,^^27^ have built upon the initial description for assessment of depth-resolved attenuation coefficient in OCT.^22^ Attenuation coefficient can be calculated based on an integral formulation in the single-scattering model outlined by Vermeer et al.,^22^ which assumes all the light is extinguished within the OCT image depth range, which is indeed in our biological samples, and the backscattered light is a fixed fraction of the attenuation coefficient. The attenuation coefficient μ at i’th pixel along a given depth profile can be represented by

$$\mu [i]=\frac{1}{2\Delta }\;\log(1+\frac{I[i]}{\sum_{i=1}^{\infty }I[i]}),$$ (1)

where I[i] is the OCT intensity at i’th pixel along the A-scan and Δ is pixel size. However, to ensure that estimations of attenuation coefficient reflect the properties of the imaged tissue, variations in the OCT intensity due to sample arm focusing and depth-dependent sensitivity are removed according to

$$I(z)=\frac{U(z)-N(z)}{S(z)},$$ (2)

where U(z) represents the power magnitude as a function of depth measured by the OCT system, N(z) is the noise floor signal, and S(z) characterizes the combined effects of sample arm focusing and depth-dependent signal decay.

We followed a number of steps to ensure that measurements across multiple imaging sessions can be compared accurately. The sensitivity of a spectral-domain OCT system as a function of depth is known to be sensitive to spectrometer alignment,^28^ and so, the alignment of the spectrometer is checked and adjusted periodically. In addition, potential variability in depth-dependent sensitivity is similarly re-assessed periodically by depth profile acquisition from a single reflector in the sample arm as the reference arm length is varied, and re-alignment of the spectrometer is performed until the depth-dependent drop-off becomes consistent with previous measurements. Another critical factor in maintaining consistency of S(z) between imaging sessions is the positioning of the focal plane within the imaging window.^22^ Prior to each imaging session, the position of a mirror was adjusted to yield maximum reflection under the sample arm scanning lens. The optical path length in the reference arm was then adjusted to position this reflection at a depth of 180  μm (100 pixels) to ensure consistency in the positioning of the focal plane. This depth is sufficiently shallow in the imaging window that samples to be imaged can be positioned below this focal plane.^29^^,^^30^ The overall signal decay curve S(z) is then measured by sequential acquisition of depth profiles as the depth of the mirror is scanned over the imaging window.

### 2.3 Experimental Procedure

**Fig. 2:**
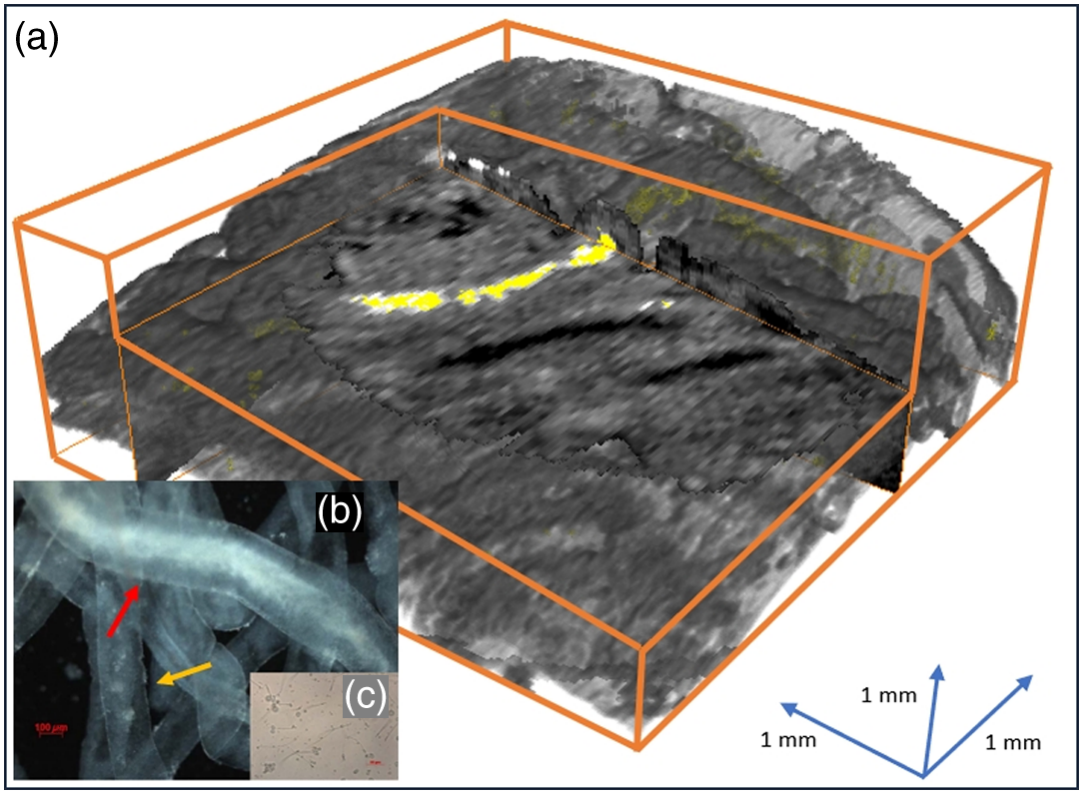
OCT and dissecting microscope with transillumination images. (a) OCT-based visualization of sperm (yellow) within the lumen of a seminiferous tubule (cropped from original volume). A block was removed to better visualize an en-face view of the sperm-bearing tubule, with a portion of a cross-sectional view of the lumen within the tubule wall. The continuation of this tubule can be seen in the region of the volume directly behind the cross-sectional view. (b) Transillumination image of the same region after removal from the testes (200×). One seminiferous tubule containing sperm is indicated by a red arrow, whereas one seminiferous tubule without sperm is indicated by a yellow arrow. (c) Microscopic image of the sperm cells from the tubules shown in panel (b).

Fifteen busulfan-treated and two non-treated control rats were used in this study. Testes were collected from rats immediately after ${\mathrm{CO}}_{2}$ inhalation euthanasia. Three very small incisions were made on the tunica albuginea over the top surface of a testis to expose small windows of seminiferous tubules (inset of Fig. 1). OCT scanning was performed in each window [Fig. 2(a)]. Immediately after scanning, the surface layers of tubules from the imaged area of each window were excised and examined by a trained morphologist under a dissecting microscope [Figs. 2(b) and 2(c)] at high magnification (200×) to determine the presence or absence of spermatozoa.^31^^–^^34^ The same procedure was then repeated after rotating the testes to expose its previously lower surface, resulting in a total of six windows per testes. A total of 180 volumetric datasets were acquired from 15 rats.

Volumetric OCT data was acquired with the following protocol. Cross-sectional images are formed from 1024 depth profiles over a lateral width of 4 mm. A volumetric data set is composed of 200 such images spanning 4 mm orthogonal to the cross-sectional image direction, resulting in OCT volumes composed of 1024×200×1024  voxels spanning $4\times 4\times 1.85\;{\mathrm{mm}}^{3}$. As previously described, efforts were taken to position the surface of all samples below the focal plane, but it should be noted that in some cases, the uneven topology of samples resulted in some portions being at or slightly above this plane. Following imaging of each volume, light in the sample arm is blocked to acquire data for assessment of the noise floor N(z). These quantifications of back-reflected OCT intensity are then processed to yield depth-resolved quantifications of the optical attenuation coefficients of the sample.^22^

### 2.4 Post-processing Procedure

OCT intensity and attenuation coefficient data were processed from raw interference information using the following procedure. An averaged spectrum obtained from the reflection in the reference arm only was subtracted from the 2048-point interference signal, followed by the application of a modified Hann window to smooth the first and last 64 pixels of the 2048-point interference signal. The resulting signals were then calibrated to k-space, and the dispersion mismatch between the sample and reference arms was compensated. A Fourier transform then yielded complex depth profiles, from which U(z) was obtained from data acquired from imaged samples and N(z) from data acquired with the reference arm alone. The calibrated intensity I(z) was then calculated using Eq. (2). A uniform filter with a kernel size of 11×10  pixels, determined based on the axial and lateral resolution of the system, was then applied to the calibrated intensity frame to reduce the effect of coherent speckle. Depth-resolved maps of the attenuation coefficient were then obtained by applying Eq. (1).

## 3 Results

### 3.1 Identification of Anatomical Features in OCT Data

**Fig. 3:**
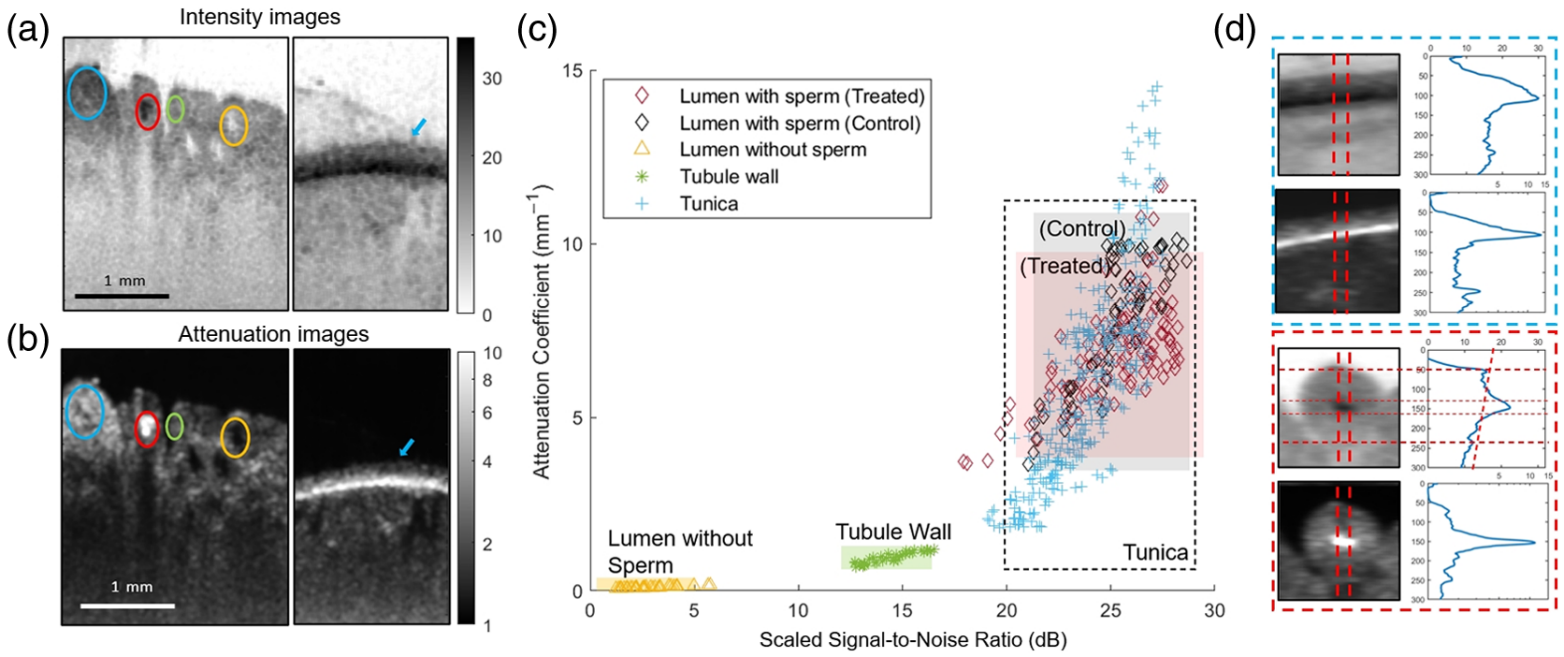
Color-coding analysis. (a) Cross-sectional OCT calibrated intensity images of, from left to right, tunica (blue), sperm-bearing lumen (red), tubule wall (green), empty lumen (yellow), and tunica (blue arrow). (b) Corresponding OCT attenuation images. (c) A scatterplot of the scaled SNR and attenuation coefficient averaged over manually segmented cross-sectional images (blue “+” indicates tunica, red “⋄” indicates lumen of sperm-bearing tubules, green “*” indicates the tubule wall, and yellow “△” indicates empty lumen). (d) Magnified cross-sectional OCT calibrated intensity and attenuation images of tunica and sperm-bearing lumen with corresponding depth profiles averaged over the area indicated between the dotted red lines.

An important next step in our study was to identify the distinct tissue types in our OCT datasets. Direct visual examination of tubules in one imaged window from a control and treated testes allowed for clear identification of a number of sperm-bearing and empty tubules, respectively, either directly before or after imaging with OCT. These same tubules, as well as portions of the tunica, were then identified in volumetric OCT data sets, as shown in Figs. 3(a) and 3(b), in which empty and sperm-bearing lumen, the tubule walls surrounding those lumen, and tunica can be clearly identified in cross-sectional images of calibrated intensity and attenuation coefficient. Regions representing the distinct structures of tunica, tubule wall, and lumens with or without sperm were then manually segmented. The scaled signal-to-noise ratio (SNR), calculated as the calibrated intensity divided by the noise floor, and attenuation coefficient within segmented regions were averaged and used to create the scatterplot shown in Fig. 3(c). The scatterplot distribution of empty lumen (${\mathrm{SNR}}_{-}=2.75\pm 1.18\;\mathrm{dB}$, $\mu_{-}=0.10\pm 0.03\;{\mathrm{mm}}^{-1}$) and tubule walls (${\mathrm{SNR}}_{\text{tubule}}=14.39\pm 1.16\;\mathrm{dB}$, $\mu_{\text{tubule}}=0.95\pm 0.16\;{\mathrm{mm}}^{-1}$) are clearly distinct regions. However, no statistically significant difference was found between the scatterplot distributions of sperm-bearing lumen from control (${\mathrm{SNR}}_{+\text{control}}=25.47\pm 1.78\;\mathrm{dB}$, $\mu_{+\text{control}}=8.24\pm 2.28\;{\mathrm{mm}}^{-1}$) and treated testes (${\mathrm{SNR}}_{+\text{treated}}=25.24\pm 2.31\;\mathrm{dB}$, $\mu_{+\text{treated}}=7.91\pm 1.46\;{\mathrm{mm}}^{-1}$). These scatterplot distributions also have significant overlap with that of the tunica (${\mathrm{SNR}}_{\text{tunica}}=23.95\pm 1.96\;\mathrm{dB}$, $\mu_{\text{tunica}}=6.18\pm 2.81\;{\mathrm{mm}}^{-1}$), indicating that clear differentiation between the sperm-bearing lumen and the tunica in the remainder of the volumetric OCT data could not be achieved purely based on the averaged calibrated intensity and attenuation coefficient values.

Although these averaged values were not sufficiently distinct, the spatial distribution of these optical characteristics of these regions reflects the vastly different morphology of sperm-bearing lumen and tunica. The tunica, being a dense fibrous tissue layer surrounding the testes, exhibits elevated calibrated intensity and attenuation coefficient over an extended depth range, as seen in the upper half of Fig. 3(d). On the other hand, sperm-bearing lumen are encapsulated by tubule wall, as seen in the lower portion of Fig. 3(d), with a smaller depth range in between exhibiting elevated calibrated intensity and attenuation coefficient. Depth profiles of the first 450  μm below the tissue surface were averaged for lateral locations with visually identified tunica and the central 50% in the lateral direction for sperm-bearing tubules to generate depth-resolved template patterns of both calibrated intensity and attenuation coefficient for these tissue types. Cross-correlation coefficients were then calculated between these template patterns and the first 450  μm of depth starting from the tissue surface. Optimized thresholds for these cross-correlation coefficients allowed for over 97.8% accuracy in the distinction between the visually identified tunica and sperm-bearing lumen.

**Fig. 4:**
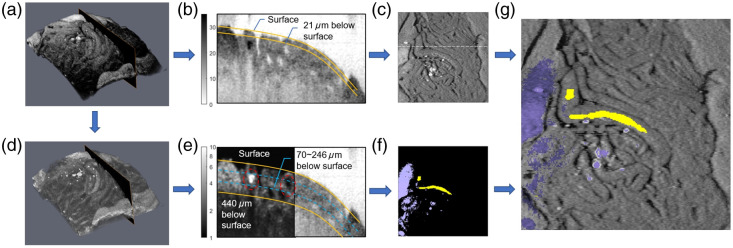
Data processing workflow. An en-face visualization of an imaged window is generated by examining a (a) volume of OCT-calibrated intensity information based on the average OCT-calibrated intensity over a (b) depth range encapsulating the upper portion of the tubule walls (21  μm below the tissue surface) surface, resulting in an en face representation (c) similar to that seen by the naked eye. The corresponding volume of (d) OCT attenuation information is then additionally used but focuses (e) on a depth range encapsulating the lumen of the surface layer of tubules (between 70 and 246  μm below the tissue surface) for an initial segmentation of tubule wall, followed by a correlation coefficient analysis over a greater depth range for identification of tunica and sperm-bearing lumen. The resulting identifications provide a (f) colormap. The two resulting en-face visualizations are combined to provide a (g) composite view in which sperm-bearing lumen can be rapidly identified. Calibrated intensity and attenuation coefficient images are displayed on a logarithmic gray scale.

We then used the following procedure for tissue type identification and visualization in all imaged datasets. The upper surface of samples was identified by locating the first point in depth in all A-lines of cross-sectional attenuation coefficient images exceeding $0.3\;{\mathrm{mm}}^{-1}$. A smoothed curve was formed using the local maxima in these surface outlines, as shown in Figs. 4(b) and 4(e). En-face representations based on the average of the scaled SNR within 21  μm of this smoothed curve provided a visualization of the surface layer of tubules within an imaged window, as seen in Fig. 4(c). This representation of only portions of the outer tubule walls is of a similar nature to that viewed by a surgeon performing micro-TESE and allowed for manual identification of the tubule within each window with the largest outer diameter. A second en-face representation was generated from the attenuation coefficient images via a maximum-intensity projection of a range of depths 70 to 245  μm below this smoothed curve [Fig. 4(e)] to identify lateral locations with the elevated calibrated intensity and attenuation coefficients consistent with either sperm-bearing lumen or tunica. A cross-correlation analysis of the first 450  μm from the tissue surface with the template patterns was performed as previously described to differentiate lateral locations corresponding to sperm-bearing lumen and tunica, as shown in Fig. 4(f). The resulting color overlay was superimposed on the en-face representation based on the scaled SNR of the overall tubule structure, as shown in Fig. 4(g). This facilitated the manual selection of the tubule with the highest average attenuation for each of the imaging windows.

### 3.2 Accuracy of Window Identification

**Fig. 5:**
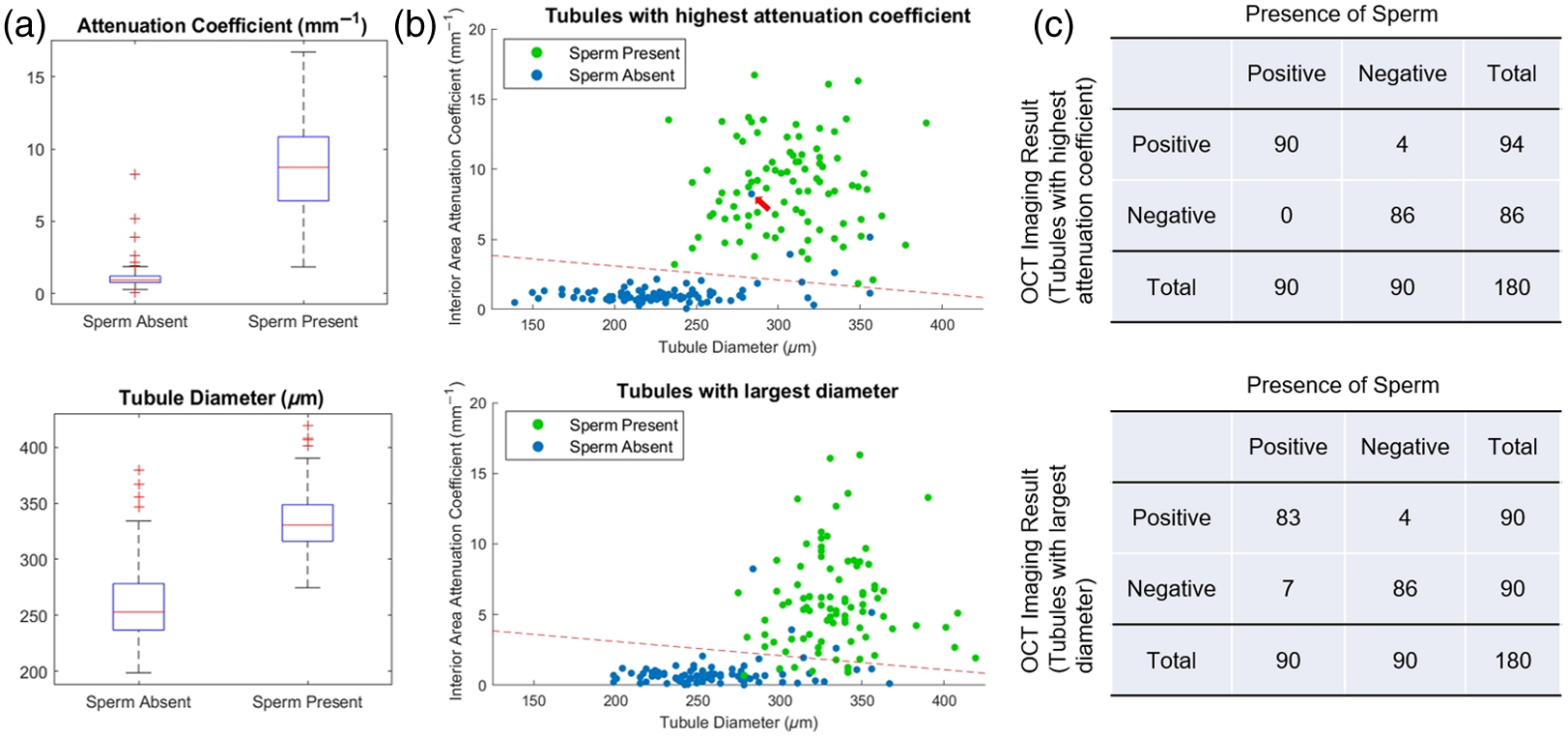
Statistical analysis. (a) Box plots showing the difference between imaged windows in which sperm was or was not visually identified for the average OCT attenuation within the lumen (top) and of the outer tubule diameter (bottom). (b) Scatterplot of outer tubule diameter versus average OCT attenuation within the lumen for all imaged windows, where representative tubules were selected based on highest average OCT attenuation (top) and largest outer tubule diameter (bottom). Threshold lines were identified to maximize overall accuracy for the identification of windows with and without sperm. (c) Corresponding classification results based on the indicated threshold lines based on tubule representation by highest attenuation coefficient (97.8% accuracy) and largest diameter (93.9%).

The overall goal of this study is to assess the accuracy with which OCT-based measures can identify whether or not a given field of view contains occult sperm. One of the primary indications currently used by surgeons is outer tubule diameter, and so, we tested three quantitative metrics: outer tubule diameter, attenuation within the lumen, and calibrated intensity within the lumen, against whether each imaged window was identified as positive or negative for the presence of sperm. Because our goal was to identify whether a given window contained even a single sperm-bearing tubule regardless of the number of empty ones, we calculated the outer tubule diameter, average lumenal attenuation coefficient, and average lumenal scaled SNR for the tubules identified as previously described with the largest diameter and highest lumenal attenuation coefficient. It should be noted that the tubule with the greatest outer diameter was often not the same as the one with the highest lumenal attenuation coefficient. Figure 5(a) is a boxplot of the diameter of the thickest tubule for windows in which sperm was or was not identified by the morphologist, yielding a p-value for the difference in distributions of $p_{\text{diameter}}=0.128$. A similar analysis of the average lumenal attenuation coefficient in the tubule identified with the highest attenuation coefficient is shown in Fig. 5(b), with a p-value of $p_{\mu }=0.032$.

Figures 5(b) and 5(c) show the results using both outer tubule diameter and average lumenal optical attenuation coefficient for improved window assessment. The points in the upper scatterplot are formed from tubules chosen based on the maximum lumenal optical attenuation coefficient and based instead on tubules with the maximum outer tubule diameter in the lower one. Threshold lines were calculated for both cases to maximize overall accuracy with a bias toward minimizing false negatives to maximize detection of occult sperm. The resulting classification tables shown in Fig. 5(c) reveal these bivariate classifications yield overall accuracies of 93.9% (with a 7.8% false negative rate) and 97.8% (with a 0% false negative rate) based on selection of tubules with maximum outer tubule diameter and average lumenal attenuation coefficient, respectively.

## 4 Discussion and Conclusion

The intensity measured at a particular depth in OCT is proportional to both the local reflectivity of the sample, to the intensity of light reaching that location, as well as depth-dependent sensitivity resulting from sampling of the OCT interference signal with a finite spectral bandwidth^28^ and sample arm focusing effects.^30^ As a result, measures of the intensity back-reflected from similar structures at one location can differ from those in a different location in the OCT imaging window. This likely hampered the detailed quantification of the observation in Trottmann et al. of the elevated characteristic OCT reflections of seminal fluid in a bovine animal model.^16^ We observe similarly elevated levels of OCT intensity in the lumen of sperm-bearing tubules in our murine model. Determination of the depth-resolved attenuation coefficient in OCT not only provides quantification of the local optical attenuation of the tissue but also in a manner invariant to the tissue above it and with demonstrated removal of other common artifacts.^22^ This allows for enhanced robustness with which the elevated optical attenuation of sperm^35^ can be detected over previous studies that utilized only the base intensity information provided by OCT. Combining both calibrated intensity and attenuation coefficient values enables efficient tissue differentiation in our study, as illustrated in Fig. 3(c).

**Fig. 6:**
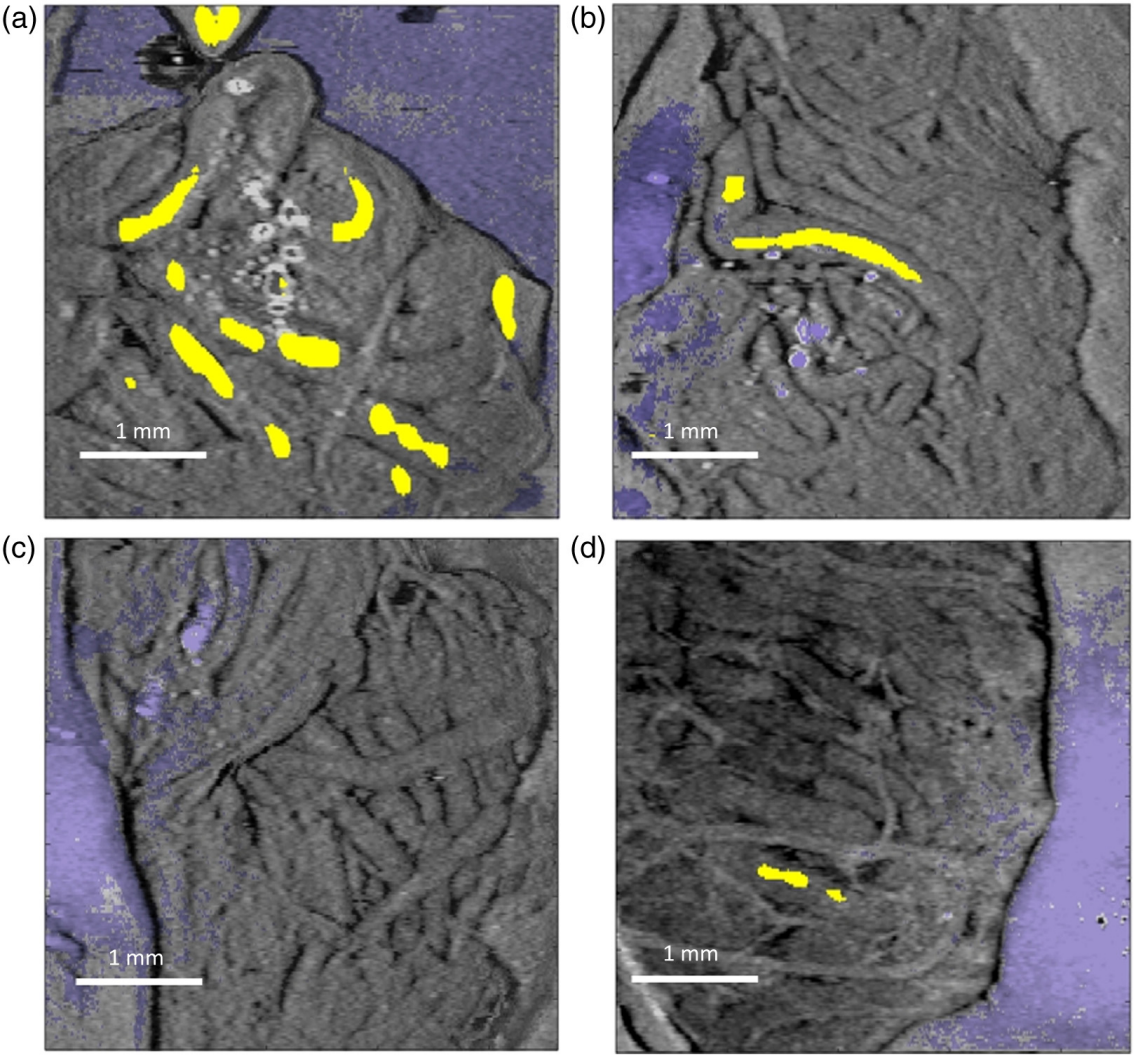
Color-coded and template-matching results. Example composite visualization from imaged windows color-coded to rapidly identify tunica (blue), tubule wall, and lumen containing sperm (yellow) from (a) the testes of a control animal, (b) treated testes in which sperm was identified, and (c) treated testes in which no sperm was identified. (d) Depicts a similar view for the imaged window highlighted by the red arrow in Fig. 5(b), for which sperm was not visually identified but where a small region with optical characteristics similar to those of sperm-bearing tubules can be seen.

Our results demonstrated a high degree of correlation between OCT-based determination of whether an imaged region contained a sperm-bearing tubule and examination under a light microscope for the presence or absence of sperm. As a first step toward using OCT for surgical guidance, the procedure described earlier to generate Fig. 4(g) can be used to potentially identify exactly which tubule(s) contain sperm. Examples of imaging windows from control and treated testes, with and without observation of sperm, are shown in Fig. 6, allowing for facilitated identification of individual tubules likely to contain sperm as seen in the representative images in Fig. 6 from an imaged window from the testes of a control animal [Fig. 6(a)], that of treated testes in which sperm was identified [Fig. 6(b)], and when sperm was not [Fig. 6(c)].

Figure 6(d) shows the en-face view of the window highlighted by the red arrow in Fig. 5(b). The corresponding calibrated intensity, attenuation coefficient, and tubule diameter are from the tubule marked in yellow. This window is notable for having a tubule with an elevated average lumenal attenuation coefficient despite no observation of sperm under direct examination. Although it is entirely likely that this can be simply attributed to a false positive, there is a possibility that this represents a more exciting situation: a case where gross visualization examination of a sample of tissue missed a small pocket of sperm that OCT-based visualization was able to detect. We plan to investigate this more tantalizing possibility in the near future.

The results here represent the first demonstration, to the best of our knowledge, of the utility of OCT attenuation for detecting the presence or absence of sperm within imaged windows for an animal model of NOA. Statistical analysis based on the selection of tubules with the largest diameter for the presence or absence of sperm within a given imaging window reveals that although tubule diameter alone is capable of some discrimination between empty and sperm-bearing tubules in this rat animal model, given $p_{\text{diameter}}=0.128$, the additional consideration of the average lumenal attenuation coefficient improves the accuracy of such detection to 93.9%. Full identification of the presence or absence of sperm within an imaging window based on attenuation coefficient only slightly improves overall accuracy to 97.8%. There is a significant difference, however, between the rate of false negatives between these selection criteria; our results in this animal model indicate a 7.8% false negative rate based on the largest tubule diameter, representing tubules that likely do contain sperm but that are being misidentified. On the other hand, selection based on the attenuation coefficient had a false negative rate of 0%, a result that implies future potential for improving outcomes in micro-TESE with OCT assistance. It should be noted that we make no claims with regard to the minimum volume or cell count required for this detection; these are aspects we plan to investigate in the near future along with careful dissection and measurements on isolated seminiferous tubules to verify the identification of single sperm-bearing tubules.

## Data Availability

The raw data and statistical/analytic code pertaining to the NOA rat testis study will be provided upon request to the corresponding author. The study is funded by the National Institutes of Health and data sharing is mandatory. Requests for access to the data should be directed to the corresponding author.
